# Measuring Parental Response Styles to Child Stress in Severe Pediatric Illness: A Validation Study

**DOI:** 10.3390/nursrep14040258

**Published:** 2024-11-15

**Authors:** Carlos Pitillas, Blanca Egea Zerolo, Rafael Jódar, Ana Ribeiro

**Affiliations:** 1 University Institute of Family Studies, Comillas Pontifical University, 28049 Madrid, Spain; cpitillas@comillas.edu; 2 Health Sciences Department, San Juan de Dios School of Nursing and Physical Therapy, Comillas Pontifical University, 28049 Madrid, Spain; begea@comillas.edu; 3 San Juan de Dios Foundation, 28036 Madrid, Spain; 4 Psychology Department, Comillas University Clinic (UNINPSI), Comillas Pontifical University, 28049 Madrid, Spain; 5 Nursing Department, Faculty of Nursing, Physical Therapy, and Podiatry, Complutense University of Madrid, 28040 Madrid, Spain

**Keywords:** family relationships, invariance, parenting, pediatrics, nursing

## Abstract

Background: Pediatric illnesses not only impose physical challenges on affected children, but also profoundly impact their emotional well-being. Understanding how parents respond to their children’s psychological distress during medical experiences is crucial for enhancing the overall support provided to these families. Aim: This study evaluated the internal structure of the Parental Response Styles Questionnaire (PRSQ), designed to differentiate parental responses to psychological distress in children with pediatric illnesses. Methods: A sample of 701 parents of children with medical issues responded to the PRSQ, reporting their different emotional expressions and responses to their children’s expressions of distress during the medical experience. Results: Factor analysis confirmed, in three of the five subsamples, an internal scale structure consisting of four factors: apathy and dysphoria, irritability and rejection, overprotectiveness, and perceived maladjustment. The invariance analyses revealed that congenital heart disease and neurological disorders are more similar in function to each other than pediatric cancer. Parents of children with neurological disorders exhibited a notably insecure pattern of parental responsiveness. Conclusions: In pediatric contexts, parental responses to their children’s emotional distress are significant factors in the process of adaptation. These responses can be measured, differentiated, and, ideally, managed by nurses and other healthcare professionals. The Parental Response Styles Questionnaire (PRSQ) is a promising tool for assessing parental reactions during their children’s treatment, and its structure appears to be particularly robust across diagnoses such as pediatric cancer, congenital heart disease, and neurological disorders.

## 1. Introduction

Severe pediatric illnesses introduce many complex challenges into a child’s life that concern the medical sphere (e.g., developing new care habits, overcoming invasive treatments, and learning to live with side effects, among others) and the psychological sphere (e.g., maintaining academic and social life during treatment, developing a positive self-image, regulating difficult emotions such as fear of relapse, and integrating the illness experience into a sense of identity, among others), both during treatment and in survivorship [1,2].

The severity of the diagnosis, the intensity of the treatment, and the severity of the prognosis, among other factors, can modulate the impact a disease has on the patient’s quality of life, their psychological well-being, and the appearance of symptoms of depression, anxiety, or posttraumatic stress [3]. In addition to these variables, the emotional response and coping of the patient’s family may be relevant in determining the level of psychological adaptation of the child to the disease. Several studies suggest that pediatric diseases such as complex congenital heart disease, severe neurological diseases, and cancer can be very relevant stressors for other family members and the family system as a whole [4,5,6]. The diagnosis abruptly disrupts and changes family dynamics for many patients and their families. Family members must cope with medical consultations, frequent complex medical exams, hospital stays, and possibly surgeries [7]. It is, therefore, predictable that the diagnosis will profoundly impact the whole family [8,9]. The mental health of the parents, the parenting, the parent–child relationship, and the parent’s quality of life may be affected [10] and, in turn, affect the adjustment and psychological state of the child during treatment and survival.

For this reason, some studies have shown that parents of children with medical issues may experience higher levels of anxiety and depression and perceive more vulnerability in their children than healthy children’s parents [11,12]. Also, these parents may change some of their previous attitudes and develop patterns of overprotection [13] or see their bonding experience with the child altered in other ways [14]. Parental style is associated with the quality of adjustment and level of distress in children diagnosed with illness [15,16,17]. Also, in parents of children with medical issues, anxiety and posttraumatic stress symptoms may be relatively high [18,19] and associated with emotional problems in their children [20]. These findings are consistent with a relational perspective on posttraumatic stress in the pediatric context [21,22,23] and, more generally, with a family-centered view of psychosocial adjustment in pediatric patients [24].

The success of comprehensive pediatric care depends, for the most part, on our ability to accurately assess parental responses to patient distress derived from their medical experience. Despite previous efforts in this direction [25,26,27], to our knowledge, there are no validated measures that probe parental responses in pediatric settings with high specificity and discrimination (e.g., differentiating between overprotective and critical responses to the child).

Considering the impact of pediatric illness on parents and the importance of coping processes within the family, nursing and clinical teams need effective tools for assessing family responses to illness and identifying significant coping risks. Accurate assessment is a prerequisite for planning comprehensive care that addresses the health and psychological needs of pediatric patients and their families. In this context, this work aims to explore the internal structure of the Parental Response Styles Questionnaire [28], which was designed to assess and differentiate parental response styles in pediatric settings. Additionally, this work applies measures to evaluate response tendencies in parents of children with various serious pediatric diseases, including congenital heart disease, pediatric cancer, and neurological disorders.

## 2. Materials and Methods

### 2.1. Design and Sample

This study employed a cross-sectional survey design. A total of 701 parents of children with various pediatric conditions responded to a questionnaire regarding their responses to their children’s distress related to illness or treatment. The sample was selected using convenience sampling from healthcare settings and support organizations, with data collection occurring once.

### 2.2. Sample Scope and Selection

The sample was composed of 701 parents of children diagnosed with pediatric cancer (129 parents), congenital heart disease (141 parents), neurological disorders (90 parents), allergic disease (79 parents), or diabetes mellitus (262 parents). The participants’ mean age and the parents’ genders are shown in Table 1.

The selection of participants for the study was carried out mainly through a pediatric oncology unit and three family foundations—the Hematology and Pediatric Oncology Unit of the Hospital Universitario Madrid Montepríncipe; Fundación Menudos Corazones, Fundación AVA; and Fundación para la Diabetes—that treat these diseases or work with parents who have children with these pathologies.

Participants responded within two different time frames. In the congenital heart disease, neurological disorders, allergic disease, and diabetes mellitus samples, parents were informed about their current response style. For parents with children suffering from a chronic condition, their children’s medical experience was taking place at the same time as the study, and the parental responses evaluated by the PRSQ were about the patients’ current distress. Alternatively, in the cancer sample, we studied responses from parents whose children had survived the disease and whose treatment had ended months or years ago. In this case, parents responded to the PRSQ thinking about their reaction to the child’s distress in the past. Despite measurement bias related to this time-lapse (see below for a discussion on this), we conducted measurements within these two time frames to ascertain the questionnaire’s versatility.

### 2.3. Inclusion Criteria

- Parent of a pediatric cancer survivor or a patient with congenital heart disease, neurological disorders, allergic disease, or diabetes mellitus.
- Patient’s age: 0–30 years.
- Willingness to participate in the study.

Research on individuals with severe intellectual disabilities, particularly in the context of neurological disorders, demonstrates that parents often maintain intensive caregiving roles well into their children’s adulthood [29,30]. For this reason, we included subjects up to 30 years old, as they remain fully dependent on their parents for decision-making and care.

### 2.4. Data Collection and Instruments

The foundations and collaborating centers contacted the families, and a letter was sent encouraging participation in the study, explaining the research objectives, including informed consent forms and online survey links. SurveyMonkey and Gandia Qüest software (TESI, Valencia, Spain) facilitated the data collection and subsequent analysis.

As previously mentioned, questionnaires were distributed across diverse time frames for the different samples. Parents of pediatric cancer survivors were contacted and sent the measurement tool months or years after their children’s diagnosis and treatment (see below for details). In contrast, parents of children suffering from other conditions participated in the study at the same time as their children’s (chronic) conditions were affecting them.

Both parents of patients were invited to participate in the study. Families could decide which (if not both) of the caregivers responded. Surveys discriminated between the adults who played the primary or reference caregiver role during medical visits, procedures, and other activities. The present study was conducted using only responses from caregivers who played the former role, since they were the ones who spent more time with the patients during the distressing moments related to their disease.

### 2.5. The Parental Response Style Questionnaire (PRSQ)

The PRSQ [31] was used to measure the different styles of parental response in families with chronically ill children. The PRSQ-R (reduced version) [28] is an instrument that is in the process of being validated and was designed to ascertain different forms of parental response to the expressed distress of a child or adolescent in the context of a significant illness and its treatment. The instrument’s original language is Spanish, with 16 items to be answered by the patient’s father, mother, or other caregiver.

The PRSQ-R (Supplementary Materials Table S1) mainly measures four factors, to which the following items correspond:

- Factor 1: apathy/dysphoria (items 1, 2, 5, 8, 13, and 16) includes parental responses on the spectrum of depressive phenomena, such as “In any period since the onset of the illness, has it been difficult to continue taking care of yourself (e.g., continuing to do things that are pleasurable for you, grooming, resting, etc.)?”.
- Factor 2: Irritability/rejection (items 4, 10, and 11) groups parental responses of anger or discomfort towards the child or one’s parenting during the child’s treatment. For example, it includes the items “How often have you become angry with your child when the child’s complaints were too persistent?” or “How often do you notice that you cannot help but get upset at your child’s discomfort?”.
- Factor 3: Overprotection (items 6, 7, 9, and 12) includes parental responses defined by indulgence, preoccupation, and a tendency to proactively care for the child, even if the child does not need it at the present moment. For example, it includes the items “To make your child feel as good as possible, did you try to give him/her almost everything he/she wanted at every moment?” and “Have you been or are you more permissive with your child because he/she has been through too much?”.
- Factor 4: Perceived maladjustment (items 3, 5, and 14) is defined by parental difficulties in understanding the child’s distress, as well as by a tendency to make insecure assumptions about the child’s difficult behavior during treatment. For example, it includes the items “Did you find some of your child’s reactions at home or in the hospital difficult to understand?” and “Did you feel that your child’s behavior during the illness was disordered and/or unpredictable?”.

### 2.6. Data Analysis

Firstly, confirmatory factor analysis (CFA) was conducted in each sample. Following the method of Williams et al. [32], we used both liberal and conservative cutoff points for acceptable fitting of the comparative fit index (CFI), root mean square error approximation (RMSEA), non-normed fit index (NNFI), and standardized root mean square residual (SRMR). These goodness of fit indices evaluate how well the hypothesized model fits the observed data. RMSEA and SRMR assess absolute fit, while CFI and NNFI focus on comparative fit relative to a null model. The CFI and NNFI should be close to or greater than 0.90 (liberal) or 0.95 (conservative), RMSEA should be 0.10 or less (liberal) or 0.06 or less (conservative), and SRMR should be less than 0.10 (liberal) or 0.05 (conservative).

Subsequently, we conducted an invariance test across groups of patients with pediatric cancer, neurological disorders, diabetes mellitus, allergic diseases, and congenital heart disease, evaluating the models based on configural, metric, scalar, and residual invariance. The fit measurements applied included the χ^2^ statistic, chi-square ratio (χ^2^/df), CFI, Tucker–Lewis index (TLI), RMSEA, and SRMR. The cutoff criteria were set as follows: χ^2^ with a nonsignificant value (*p* > 0.05); χ^2^/df with a ratio of ≤3; CFI and TLI at ≥0.95; RMSEA at ≤0.05; and SRMR at <0.80 [33]. We assessed Δχ^2^, ΔCFI, ΔRMSEA, and ΔSRMR to gauge the goodness of fit across various levels of measurement invariance. Based on Chen’s recommendations [34], invariance was not assumed if ΔCFI ≥ −0.005, ΔRMSEA ≤ 0.010, and ΔSRMR ≤ 0.025.

We calculated Cronbach’s alpha (α) and MacDonald’s omega (ω) to estimate reliability [35]. Additionally, correlation analyses were conducted to assess convergent validity. For the CFA and invariance testing, we used M-Plus v.6, while SPSS v.28 was employed for all other analyses.

### 2.7. Ethical Considerations

This study has the approval of the ethics and research committee of the Universidad Pontificia Comillas, in addition to a favorable report by the CEIC of the Community of Madrid, Spain. The recommendations of the latest update of the Declarations of Helsinki and Tokyo of the World Medical Association have been followed. The researchers at the university adhere to the CRUE declaration on good research practice. All data and information about the study have been handled with the utmost confidentiality and in full compliance with the provisions of European Regulation 2016/679, dated 27 April 2016; the Spanish Ley Orgánica 3/2018 of 5 December, regarding the Protection of Personal Data and Guarantee of Digital Rights; and the Biomedical Research Law 14/2007 and its 2016 amendment.

## 3. Results

### Sample Description: Fathers and Mothers

Table 1 shows the descriptive parameters of the children with diagnoses of illnesses included in the study, with a total sample size of 701 participants. For neurological disorders, 90 subjects were enrolled, with a mean age of 15.77 ± 10.55 years. Of these, 37.8% were female. For congenital heart disease, 141 participants were enrolled, with a mean age of 9.23 ± 6.36 years, and 44.8% were female. For diabetes mellitus, 262 subjects were enrolled, with a mean age of 11.65 ± 4.65 years, and 48.5% were female. For pediatric cancer, 129 participants were enrolled, with a mean age of 11.90 ± 5.40 years, and 52.7% were female. Finally, 79 allergic disease participants were included, with a mean age of 7.46 ± 3.95 years, and 56.5% were male.

**Table 1:** The descriptive parameters of the subjects included in the study (n = 701).

	n	Mean Age (SD)	Gender (n Female, %)
Neurological disorders	90	15.77 (10.55)	34 (37.8%)
Congenital heart disease	141	9.23 (6.36)	64 (44.8%)
Diabetes mellitus	262	11.65 (4.65)	127 (48.5%)
Pediatric cancer	129	11.90 (5.40)	69 (52.7%)
Allergic diseases	79	7.46 (3.95)	37 (43.5%)

According to the CFA, the 16 items in the four factors model, adjusted correctly, were as follows in the subsample of pediatric cancer: (χ^2^(98) = 135.6, *p* = 0.007, RMSEA = 0.055 (90% CI = 0.029, 0.078), CFI = 0.910, TLI = 0.890, SRMR = 0.068); in congenital heart disease: (χ^2^(98) = 142.80, *p* = 0.002, RMSEA = 0.057 (90% CI = 0.035, 0.076), CFI = 0.925, TLI = 0.928, SRMR = 0.066); and in a more adjusted form, in the sample of neurological disorder patients: (χ^2^(98) = 140.5, *p* = 0.003, RMSEA = 0.071 (90% CI = 0.042, 0.097), CFI = 0.878, TLI = 0.850, SRMR = 0.074), whereas they did not achieve a good fit in the allergic diseases or diabetes mellitus subsamples (see Table 2). Tables featuring the standardized saturations of this factorial solution are shown in the Supplementary Material (Tables S2–S6).

Reliability indicators were inadequate on the overprotection subscale in all subsamples (see Table 3).

We analyzed the invariance in the three samples, which revealed an adequate fit. Model 1 was tested for configural invariance by enforcing an identical factor structure across groups without applying any constraints to the parameter estimates between them. The results for Model 1, examining configural invariance, indicate a good model fit when comparing groups with pediatric cancer and congenital heart disease (χ^2^(196) = 270.4, *p* < 0.001, RMSEA = 0.053 [90% CI = 0.036, 0.068], CFI = 0.927, TLI = 0.911, SRMR = 0.067), neurological disorders and congenital heart disease (χ^2^(196) = 275.9, *p* < 0.001, RMSEA = 0.059 [90% CI = 0.042, 0.075], CFI = 0.921, TLI = 0.903, SRMR = 0.068), and neurological disorders and pediatric cancer (χ^2^(196) = 276.7, *p* < 0.001, RMSEA = 0.061 [90% CI = 0.044, 0.077], CFI = 0.903, TLI = 0.881, SRMR = 0.070).

Model 1 served as the baseline for assessing metric invariance in Model 2. Table 4 presents the comparison of the invariance models. In this model, factor loadings were constrained to make them identical across samples. However, the fit indices indicated inadequate adjustment for pediatric cancer vs. congenital heart disease (χ^2^(208) = 320.21, *p* < 0.001, RMSEA = 0.063 [90% CI = 0.049, 0.077], CFI = 0.890, TLI = 0.873, SRMR = 0.090) and neurological disorders vs. pediatric cancer (χ^2^(208) = 311.4, *p* < 0.001, RMSEA = 0.067 [90% CI = 0.051, 0.082], CFI = 0.875, TLI = 0.856, SRMR = 0.087). Model 2 showed a decline in fit compared to the configural model in pediatric cancer vs. congenital heart disease (∆χ^2^ (12) = 49.98, *p* < 0.001, ΔCFI = −0.037) and neurological disorders vs. pediatric cancer (∆χ^2^ (12) = 36.96, *p* < 0.001).

On the other hand, for neurological disorders vs. congenital heart disease, Model 2 displayed adequate adjustment indexes (χ^2^(208) = 287.8, *p* < 0.001, RMSEA = 0.058 [90% CI = 0.040, 0.073], CFI = 0.921, TLI = 0.909, SRMR = 0.080). Model 2 did not show a decline in fit compared to the configural model (∆χ^2^ (12) = 15.02, *p* = 0.241).

The scalar invariance model (Model 3), in which item intercepts were constrained to make them equal for neurological disorders and congenital heart disease groups, demonstrated an acceptable fit (χ^2^(220) = 302.24, *p* < 0.001, RMSEA = 0.057 [90% CI = 0.040, 0.072], CFI = 0.919, TLI = 0.911, SRMR = 0.085) and showed no deterioration in fit (∆χ^2^ = 15.38, *p* = 0.221).

Lastly, the residual invariance model (Model 4) did not achieve the desired fit (χ^2^(236) = 360.91, *p* < 0.001, RMSEA = 0.065 [90% CI = 0.053, 0.081], CFI = 0.876, TLI = 0.874, SRMR = 0.132) and showed a significantly poorer fit compared to the scalar invariance model (∆χ^2^ = 62.66, *p* < 0.001).

The means in the sample of parents with children with neurological disorders were significantly higher than those of the congenital heart disease sample for all four factors: apathy/dysphoria (difference = 0.844, SE = 0.17, *p* < 0.001), irritability/rejection (difference = 0.427, SE = 0.18, *p* = 0.015), perceived maladjustment (difference = 0.819, SE = 0.15, *p* < 0.001), and overprotection (difference = 0.514, SE = 0.17, *p* = 0.003).

## 4. Discussion

This study aimed to explore the internal structure of the PRSQ [28,31], an instrument that aims to detect some of the most common insecure parental responses to child distress in the pediatric setting, with particular attention to serious pediatric illnesses. For this purpose, the PRSQ was administered to a sample of 701 parents of children diagnosed with pediatric cancer, congenital heart disease, neurological disorders, allergic disease, or diabetes mellitus.

The factor analysis results confirmed an internal structure of the scale consisting of four factors, comprising different aspects of insecure parental response: apathy and dysphoria, irritability and rejection, overprotection, and perceived maladjustment. This structure was confirmed in three of the five research subsamples (pediatric cancer, congenital heart disease, and neurological disorders). The scale’s internal structure for these subsamples is probably stable because these three diagnoses share relevant aspects in terms of their psychological dimension. These are conditions that seriously jeopardize the survival of the children or their physical integrity, introduce high doses of daily stress in the families and an experience of mourning for the lost “normality”, and inflict substantial burdens on families’ organization and function [12,14,36]. In contrast, allergic diseases and diabetes mellitus, while sharing some aspects of this psychological phenomenology, may have a more diluted impact over time, presenting a less dramatic experience of the grief and fears associated with the diagnosis, and a less negative cultural representation than the former.

Our results converge with evidence from previous studies [4,5,6,7,8,9,10] which shows that pediatric disease introduces significant stressors into the family life and may stimulate changes in family function that range from adaptation to negative responses and/or posttraumatic reactions in the long term. By enabling a precise assessment of what parents do when their children show medical issue-related distress, the PRSQ may be instrumental in relating specific patterns of family response to differences in quality of life, family and patient adjustment to disease, and long-term consequences. Moreover, our study adds a focus on parental insecure responses that go beyond anxiety and overprotection, which have been the most widely studied [11,12,13,14]. Parental irritability/rejection and parents perceiving their children’s responses as symptoms of maladjustment are aspects of parental response that are initially measured by the PRSQ. They may have specific significance to both parents’ and patients’ adjustment processes, as well as in the meaning-making and interpersonal exchanges that influence the development of posttraumatic suffering in children with medical issues [25].

The invariance results indicate that congenital heart disease and neurological disorders function more similarly to each other than pediatric cancer, which also seems to point to differences in the family experience of these diseases. Although they can be significantly prolonged, pediatric cancer treatments are more concentrated in time than treatments for congenital heart disease and neurological disorders. For an average of two and a half years (if there are no relapses), children with cancer and their parents are exposed to a continuous succession of stressful events, invasive procedures, and very painful treatment side effects [37]. Congenital heart disease and neurological disorders, although also punctuated by occasional events of great physical and emotional distress (e.g., surgeries in congenital heart disease), involve a more chronic disease experience than cancer, with different implications in terms of buffering against stress, but also in terms of the psychological burden accumulated in the long term. These discrepancies could be associated with different parental responses, which could also explain the other results in terms of the invariance between these three subsamples. Future research, with more extensive and diverse samples, may help clarify these hypotheses.

Another hypothesis that may help explain these differences has to do with the time frame of the measurement. For parents in the pediatric cancer group whose children had been diagnosed and treated years earlier, the scale items were formulated in the past tense (e.g., “Did you find it difficult to conceal your worry about your child?”). For the other diseases, these questions were conjugated in the present tense (“Do you find it difficult to conceal your worry about your child?”). This alteration may have introduced a bias in how parents in the pediatric cancer group assess their own response to the distress of a child whose treatment ended years ago. A possible tendency of participants in this subsample may have been to recall only the most serious situations that were perceived as negative aspects of the treatment process, which may limit the results.

It is also important to highlight that the chronic stress experienced by this cancer subgroup has ended, as they are now survivors. In this scenario, differences in tense and the influence of memory may account for the observed variations. This limitation stems from our interest in studying our measurement tool’s ability to provide reliable results in different formats and time frames. In the future, assessment of pediatric cancer patients’ parents before and after the survival phase and study designs that consider both the temporal distance from treatment and the potential impact of memory bias on retrospective assessments will enable us to ascertain the PRSQ’s ability to provide stable measurement of parental response style over time.

The overprotection subscale presented low levels of reliability in all subsamples. Notably, items 7 and 9 seem to be the most implicated in this result. These items may be problematic in at least two ways. On the one hand, the discriminative ability of item 9, due to its formulation, may be very low. Although the item is intended to reflect the parents’ intention to prevent any form of child distress (and, therefore, to represent a manifestation of overprotection), the statement “You want to do everything possible to reduce your child’s distress” is one to which any parent would most likely agree. On the other hand, item 7 (“When another child is having a hard time, do you try not to let your child notice?”) may assess an aspect of the experience that is particularly relevant to pediatric cancer and perhaps not very applicable to evaluating the experiences of parents with other illnesses. Patients with this diagnosis spend a great deal of time in hospital or undergoing outpatient treatment, which exposes them to ongoing contact with other patients’ stress and, in some cases, death. In this context, attempts to distract the child’s attention are a relevant aspect of the parental response, but not necessarily for parents of children with congenital heart disease and neurological disorders.

The final limitation is the potential for social desirability bias, as parents may hesitate to express difficult emotions or responses (e.g., anger) toward their children to the professionals involved in their care. Future research using this instrument should aim to control this effect as much as possible.

Regarding the response patterns observed in the different subsamples, the results suggest that neurological disorders may have a greater impact on parents than the other diseases studied. These parents report feeling more dysphoria or apathy due to their child’s illness and distress, being more irritable with their child, being more overprotective, or perceiving a mismatch in their child’s responses more frequently than parents of other patients. These feelings could be in response to the greater complexity of these illnesses and the associated emotional experience. The subsample of neurological disorder patients is composed of children and adolescents with organic pathologies that concur with intellectual disability and severe behavioral problems [38,39]. It is plausible that these parents are overloaded with more grief that is difficult to process compared to families facing other illnesses and diagnoses. Unfortunately, our current results do not allow us to reach such conclusions, and more detailed research is warranted to better understand families’ experiences with each disease over time. These hypotheses should be investigated in the future, using a combination of quantitative methodology (such as that offered by the PRSQ) and a qualitative exploration of the illness experience.

## 5. Conclusions

The PRSQ was found to have a robust and stable internal structure in a significant number of subsamples of parents of children diagnosed with severe pediatric illnesses.

The results indicate that the assessment of parental responses in families affected by pediatric cancer, congenital heart disease, and neurological disorders can benefit from this efficient and applicable instrument used by a wide range of professionals. Comparison of the results of this questionnaire between parents of children with congenital heart disease and neurological disorders is also possible, as revealed by invariance analyses.

Obtaining data on the dysfunction of parental responses, both at a general level and discriminating between different forms of response, can be a helpful tool in understanding the adaptive processes of each family and planning individualized psychosocial assistance. Likewise, this instrument can provide an impetus for research into the influence of the family on processes traditionally associated with the patient’s psychosocial adjustment (e.g., quality of life or posttraumatic stress). Finally, the PRSQ could be a valuable tool for nurses and other clinical staff to assess and support families. Practitioners could use it to evaluate how families cope with the diagnosis and treatment of pediatric illness and determine the degree of risk, enabling them to plan individualized support for parents and patients. Additionally, within the framework of a well-constructed supportive or therapeutic relationship, this instrument could help parents of children with pediatric illnesses recognize their common insecure response tendencies and, with guidance, develop more adaptive strategies for coping with their children’s illnesses.

## Figures and Tables

**Table 2:** CFA four factors model fit indices, MLR estimation.

		Model Fit						
	n	χ^2^	df	χ^2^/df	CFI	TLI	RMSEA (90% CI)	SRMR
Neurological disorders	90	140.5	98	1.4	0.878	0.850	0.071 (0.042–0.097)	0.074
Congenital heart disease	141	142.8	98	1.5	0.925	0.928	0.057 (0.035–0.076)	0.066
Diabetes mellitus	262	264.5	98	2.7	0.859	0.827	0.081 (0.069–0.092)	0.063
Pediatric cancer	129	135.6	98	1.4	0.910	0.890	0.055 (0.029–0.078)	0.068
Allergic diseases	79	179.0	98	1.8	0.787	0.739	0.102 (0.078–0.126)	0.079

**Table 3:** Cronbach’s alpha and McDonald’s omega for each subscale and sample.

	Apathy/ Dysphoria		Irritability/ Rejection		Perceived Maladjustment		Overprotection	
	α	ω	α	ω	α	ω	α	ω
Neurological disorders	0.795	0.801	0.719	0.731	0.795	0.810	0.546	0.593
Congenital heart disease	0.785	0.796	0.785	0.789	0.663	0.708	0.607	0.642
Diabetes mellitus	0.806	0.800	0.685	0.690	0.618	0.623	0.669	0.684
Pediatric cancer	0.769	0.777	0.739	0.753	0.536	0.554	0.449	0.493
Allergic diseases	0.832	0.838	0.760	0.768	0.580	0.600	0.436	0.534

**Table 4:** Comparisons of invariance measurements between models.

	LL	Parents	∆χ^2^	*p*	∆CFI	∆RMSEA	∆SRMR
**Pediatric vs. congenital heart disease**							
Model 1 configural invariance	−4456.84	108	-	-			
Model 2 vs. Model 1 Metric invariance	−4481.84	96	49.98	<0.001	−0.037	0.01	0.023
**Neurological disorders vs. pediatric cancer**							
Model 1 configural invariance	−3756.27	108	-	-			
Model 2 vs. Model 1 Metric invariance	−3774.75	96	36.96	<0.001	−0.028	0.006	0.017
**Neurological disorders vs. congenital heart disease**							
Model 1 configural invariance	−3706.97	108	-	-			
Model 2 vs. Model 1 Metric invariance	−3714.47	96	15.02	0.241	0	−0.001	0.012
Model 3 vs. Model 2	−3722.16	84	15.38	0.221	−0.002	−0.001	0.005
Scalar invariance	−3753.49	68	62.66	<0.001	−0.043	0.011	0.047

## Data Availability

The original contributions presented in the study are included in the article/Supplementary Materials, further inquiries can be directed to the corresponding authors.

## Supplementary Materials

The following supporting information can be downloaded at: https://www.mdpi.com/article/10.3390/nursrep14040258/s1, Table S1: Parental Response Styles Questionnaire (PRSQ-R); Table S2: Pediatric cancer sample. Standardized saturations (and standard errors); Table S3: Allergic diseases sample. Standardized saturations (and standard errors); Table S4: Neurological disorders sample. Standardized saturations (and standard errors); Table S5: Congenital heart disease sample. Standardized saturations (and standard errors); Table S6: Diabetes mellitus sample. Standardized saturations (and standard errors).

## Public Involvement Statement

There was no public involvement in any aspect of this research.

## Guidelines and Standards Statement

This manuscript was drafted using the STROBE Statement for observational studies.
