# Supplementary material for: Measuring Parental Response Styles to Child Stress in Severe Pediatric Illness: A Validation Study
Source: Nurs Rep. 2024 Nov 15;14(4):3539–49. doi: 10.3390/nursrep14040258 (PMC11587408; doi:10.3390/nursrep14040258)
Supplement: Supplementary file 1 [file nursrep-14-00258-s001.zip › nursrep-3230947-supplementary.pdf]

Supplementary material

Table S1. Parental Response Styles Questionnaire (PRSQ-R).

Factor	Name	Item number	Item
1	Apathy/dysphoria	15	At any period since the onset of the illness, are you finding it difficult to continue taking care of yourself (e.g., doing things pleasurable for you, getting ready, resting, etc.)?
		16	Are there times when your attention to your child's medical issues (symptoms, medications...) takes time away from having fun with your child?
		13	Since the illness, do you notice at any time that you feel less inclined to do the things you used to want to do?
		1	Does your child see you having a hard time because of the disease?
		8	Do you find it difficult to hide your concern in front of your child?
		2	How often do you show sadness in front of your child for any reason?
2	Irritability	4	How often do you lose your patience slightly when your child is upset?
		10	How often do you become angry with your child when the child's complaints are too persistent?
		11	How often do you notice that you can't help but get upset at your child's discomfort?
4	Perceived maladjustment	3	Do you find it difficult to understand some of your child's reactions at home or in the hospital?
		5	Is comforting your child when he/she is having a hard time a difficult task?
		14	Do you feel your child's behavior is disorderly and/or unpredictable?
3	Overprotection	6	Have you been or are you more permissive with your child because he/she is going through too much?
		12	To make your child feel as good as possible, do you try to give him/her almost everything he/she wants at all times?
		9	Do you want to do everything possible to reduce your child's discomfort?
		7	When another child is having a hard time, do you try not to let your child notice?

Table S2. Pediatric cancer sample. Standardized saturations (and standard errors).

	Apathy/dysphoria		Irritability/rejection		Perception of maladjustment		Overprotection	
C15	0.565	(0.082)						
C16	0.431	(0.095)						
C13	0.532	(0.092)						
C1	0.684	(0.067)						
C8	0.700	(0.072)						
C2	0.683	(0.074)						
C4			0.850	(0.058)				
C10			0.709	(0.057)				
C11			0.548	(0.096)				
C3					0.707	(0.085)		
C5					0.435	(0.097)		
C14					0.470	(0.104)		
C6							0.659	(0.098)
C12							0.734	(0.078)
C9							*-0.037*	*(0.090)*
C7							0.266	(0.102)

Saturations that are not statistically significant are in italics (item C9).

Table S3. Allergic diseases sample. Standardized saturations (and standard errors).

	Apathy/dysphoria		Irritability/rejection		Perception of maladjustment		Overprotection	
C15	0.723	(0.097)						
C16	0.698	(0.107)						
C13	0.723	(0.095)						
C1	0.585	(0.108)						
C8	0.695	(0.075)						
C2	0.655	(0.102)						
C4			0.749	(0.083)				
C10			0.537	(0.108)				
C11			0.823	(0.063)				
C3					0.531	(0.120)		
C5					0.577	(0.114)		
C14					0.598	(0.086)		
C6							0.660	(0.121)
C12							0.704	(0.134)
C9							*0.172*	*(0.164)*
C7							*0.233*	*(0.171)*

Saturations that are not statistically significant are in italics (items C7 and C9)

Table S4. Neurological disorders sample. Standardized saturations (and standard errors).

	Apathy/dysphoria		Irritability/rejection		Perception of maladjustment		Overprotection	
C15	0.709	(0.096)						
C16	0.738	(0.085)						
C13	0.665	(0.079)						
C1	0.640	(0.108)						
C8	0.276	(0.136)						
C2	0.533	(0.124)						
C4			0.800	(0.087)				
C10			0.496	(0.146)				
C11			0.605	(0.117)				
C3					0.834	(0.048)		
C5					0.604	(0.084)		
C14					0.752	(0.070)		
C6							0.782	(0.134)
C12							0.616	(0.115)
C9							0.409	(0.132)
C7							*0.139*	*(0.121)*

Saturations that are not statistically significant are in italics (item C7)

Table S5. Congenital heart disease sample. Standardized saturations (and standard errors).

	Apathy/dysphoria		Irritability/rejection		Perception of maladjustment		Overprotection	
C15	0.645	(0.070)						
C16	0.802	(0.044)						
C13	0.699	(0.059)						
C1	0.624	(0.066)						
C8	0.594	(0.060)						
C2	0.409	(0.094)						
C4			0.721	(0.061)				
C10			0.692	(0.058)				
C11			0.815	(0.069)				
C3					0.734	(0.068)		
C5					0.572	(0.081)		
C14					0.622	(0.084)		
C6							0.832	(0.073)
C12							0.690	(0.074)
C9							0.294	(0.100)
C7							0.326	(0.118)

Table S6. Diabetes mellitus sample. Standardized saturations (and standard errors).

	Apathy/dysphoria		Irritability/rejection		Perception of maladjustment		Overprotection	
C15	0.695	(0.050)						
C16	0.676	(0.045)						
C13	0.735	(0.039)						
C1	0.602	(0.058)						
C8	0.579	(0.052)						
C2	0.561	(0.068)						
C4			0.577	(0.060)				
C10			0.657	(0.048)				
C11			0.717	(0.051)				
C3					0.618	(0.056)		
C5					0.562	(0.053)		
C14					0.614	(0.051)		
C6							0.722	(0.044)
C12							0.790	(0.044)
C9							0.460	(0.071)
C7							0.440	(0.055)
